# MTAP and p16 IHC as Markers for CDKN2A/B Loss in Meningiomas

**DOI:** 10.3390/cancers16193299

**Published:** 2024-09-27

**Authors:** Hanim I. Ozkizilkaya, Anjali Vinocha, Antonio Dono, Oluwaseun Basit Ogunbona, Gokce A. Toruner, Phyu P. Aung, Carlos Kamiya Matsuoka, Yoshua Esquenazi, Franco DeMonte, Leomar Y. Ballester

**Affiliations:** 1 Division of Pathology and Laboratory Medicine, The University of Texas MD Anderson Cancer Center, Houston, TX 77030, USA; hanimistem.ozkizilkaya@med.usc.edu (H.I.O.); avinocha@mdanderson.org (A.V.); ogunbona@uthscsa.edu (O.B.O.); gatoruner@mdanderson.org (G.A.T.); paung@mdanderson.org (P.P.A.); 2 Vivian L. Smith Department of Neurosurgery, The University of Texas, Health Science Center at Houston, Houston, TX 77030, USA; antonio.dono@uth.tmc.edu (A.D.); yoshua.esquenazilevy@uth.tmc.edu (Y.E.); 3 Department of Neuro-Oncology, The University of Texas MD Anderson Cancer Center, Houston, TX 77030, USA; ckamiya@mdanderson.org; 4 Center for Precision Health, School of Biomedical Informatics, The University of Texas Health Science Center at Houston, Houston, TX 77030, USA; 5 Memorial Hermann Hospital-TMC, Houston, TX 77030, USA; 6 Department of Neurosurgery, The University of Texas MD Anderson Cancer Center, Houston, TX 77030, USA; fdemonte@mdanderson.org

**Keywords:** MTAP, meningioma, *CDKN2A*, *CDKN2B*, p16, IHC, FISH

## Abstract

**Simple Summary:**

Cyclin-dependent kinase inhibitor 2A/B (CDKN2A/B) loss is a key factor in diagnosing meningiomas as Central Nervous System (CNS) WHO grade 3 tumors. Typically, detecting this gene loss involves costly and complex techniques like sequencing or FISH. However, the MTAP gene, which is located near CDKN2A/B on the same chromosome, may provide a more accessible way to detect these losses through a simpler, more affordable test called immunohistochemistry (IHC). This study explored different concentrations and antibodies for MTAP and p16 across two institutions to evaluate their potential as surrogate markers for CDKN2A/B loss. The results showed that p16 expression varied and did not align with either MTAP expression or *CDKN2A* FISH results. This study suggests that IHC for MTAP is a promising, cost-effective method for identifying high-grade meningiomas, offering a quicker and less expensive alternative to existing techniques.

**Abstract:**

Background: Homozygous cyclin-dependent kinase inhibitor 2A/B (CDKN2A/B) loss is one of the parameters that support the designation of meningiomas as Central Nervous System (CNS) WHO grade 3 tumors. Evaluation of CDKN2A/B by sequencing or Fluorescence in situ hybridization (FISH) is costly and not always readily accessible. An immunohistochemistry (IHC)-based marker for the evaluation of CDKN2A/B loss would provide faster results at a lower cost. Methods: This retrospective study included patients diagnosed with meningioma at our institution between 2016 and 2019. Archival tumor tissue was used for analysis. MTAP immunohistochemistry (IHC) was performed at various dilutions (1:1200, 1:400, 1:200, 1:100) using two different antibodies, and p16 IHC was conducted simultaneously. These analyses were carried out at two different institutions. To determine the sensitivity and specificity of MTAP and p16 as surrogate markers for CDKN2A/B loss, *CDKN2A* FISH was utilized as the gold standard. Results: Overall, 46/49 tumors showed strong MTAP staining (94%) at institution 1, and 44/49 (90%) showed either faint positive or positive results at institution 2. One grade 3 meningioma that demonstrated homozygous *CDKN2A* loss by FISH also showed loss of MTAP expression by IHC. One grade 2 meningioma showed regional *CDKN2A* loss by FISH and variable MTAP expression under different IHC conditions. MTAP expression evaluation was superior at a dilution of 1:100 with the Abnova Anti-MTAP Monoclonal antibody. Conclusions: P16 expression was variable and did not correlate with either MTAP expression or *CDKN2A* FISH results. MTAP IHC is a promising surrogate marker for the evaluation of *CDKN2A* status in meningiomas.

## 1. Introduction

Meningiomas are the most common primary Central Nervous System (CNS) tumors (39%) with a higher incidence in females compared with males [1]. While histologic features are an important part of the World Health Organization (WHO) grading for meningiomas, some molecular alterations (such as mutations in SWI/SNF-Related, Matrix-Associated, Actin-Dependent Regulator of Chromatin, Subfamily E, Member 1 (SMARCE1), BRCA1-Associated Protein-1 (BAP1), and Telomerase Reverse Transcriptase (TERT) promoters) are relevant as part of the subtyping and grading criteria [2]. In addition, Cyclin-dependent kinase inhibitor 2A/B (CDKN2A/B) homozygous deletion is one of the molecular alterations that support CNS WHO grade 3 designations for meningiomas [2]. Therefore, it is important to identify meningiomas with CDKN2A/B homozygous loss for the accurate risk stratification of patients. CDKN2A/B homozygous loss can be detected by Fluorescence in situ hybridization (FISH), Next-Generation Sequencing (NGS), or the Comparative Genomic Hybridization array (CGH-array) [3]. However, these methods are more expensive than immunohistochemistry (IHC) and are not readily available at every institution. Therefore, a reliable IHC-based assay to detect CDKN2A/B homozygous loss in meningiomas will improve clinical practice by reducing the cost and turn-around time of diagnosing and grading meningiomas according to the WHO criteria [2].

The *CDKN2A* gene is located on the short arm of chromosome 9 (9p21) and encodes the protein p16INK4a or INK4a (inhibitor of kinase 4a) [4]. *CDKN2B* is a homologous gene that encodes the protein p15INK4b or INK4b and is located approximately 20 to 30 Kbp upstream [4]. *CDKN2A* (p16) is a tumor suppressor gene that competitively inhibits cyclin-dependent kinase4 and plays significant roles in the p53 and RB1 cell cycle pathways [4,5].

S-methyl-5′-thioadenosine phosphorylase (MTAP) is a tumor suppressor gene located on chromosome 9p21, adjacent to the *CDKN2A* and *CDKN2B* genes [6]. It encodes a rate-limiting metabolic enzyme that plays a significant role in polyamine and purine metabolism [6]. MTAP loss is associated with *CDKN2A* deletion in the 9p21 locus [7]. MTAP deletion is seen in approximately 15% of all solid tumors such as glioblastoma, pleural mesothelioma, and non-small cell lung cancers [8]. Loss of MTAP expression and *CDKN2A* homozygous deletion (detected by FISH) could be helpful in pleural mesothelioma diagnosis [9,10]. Recently, it has been suggested that MTAP expression by IHC can be utilized as a surrogate marker for CDKN2A/B loss in meningiomas [11]. However, additional studies are needed to confirm the utility of MTAP expression for the diagnosis and grading of meningiomas. In this study, we evaluate the utility of evaluating p16 and MTAP expression by IHC as surrogate markers for homozygous deletion of CDKN2A/B in meningiomas. The hypothesis of this study is that MTAP and p16 expression as assessed by IHC can act as reliable surrogate markers for detecting homozygous CDKN2A/B loss in meningiomas, providing a cost-effective alternative to more expensive and less accessible molecular techniques.

## 2. Materials and Methods

Patient cohort: This retrospective study included patients diagnosed with meningioma at our institution between 2016 and 2019. Archival tumor tissue was used for analysis. Electronic medical records were screened, and a total of 50 patients with available formalin-fixed paraffin-embedded (FFPE) tissue were included in this study. The cohort consisted of 26 meningiomas WHO grade 1, 16 meningiomas WHO grade 2, and 8 meningiomas WHO grade 3. Demographics, histologic features (mitotic count, Ki67 proliferation index), and follow-up data were obtained from electronic medical records.

Tissue Microarray: One representative FFPE block from each patient was selected for inclusion in a tissue microarray (TMA). Tumor areas were marked in Hematoxylin and Eosin (H&E)-stained glass slides and 2 mm punches were performed from the corresponding areas in the FFPE tumor blocks. The tissue cores were inserted in a mold for TMA construction.

IHC: IHC for MTAP and p16 was performed in two different Clinical Laboratory Improvement Amendments (CLIA)-certified and College of American Pathologists (CAP)-accredited laboratories at 2 different institutions. Tissue sections (4 µm) were generated from the TMAs and deparaffinized using xylene, followed by rehydration. Antigen retrieval was performed using a Dako PT Link with a pH 9.0 buffer at 99 °C for 20 min. For MTAP expression, tissue sections were incubated with anti-MTAP antibody [Abnova Anti-MTAP Monoclonal (2G4), Catalog # H00004507-M01, dilution 1:400, 1:200 and 1:100 (Abnova, Taipei, Taiwan) at institution #1 and Protein Tech Anti-MTAP Polyclonal, 11475-1-AP, dilution 1:1200, (Proteintech, Rosemont, IL, USA) at institution #2]. For p16 expression, tissue sections were incubated with an anti-p16 antibody [Roche cat. 705-4793 clone E6H4 Ready-to-Use Antibody performed on the Dako Omnis (Agilent Technologies Inc., Santa Clara, CA, USA) at institution #1, and Roche cat. 06680003001 clone E6H4 on the BenchMark ULTRA system at institution #2] for 30 min at room temperature. MTAP and p16 expressions were evaluated by a board-certified neuropathologist (LYB) and scored as positive, faint positive, focal positive, or negative.

### Fluorescent In Situ Hybridization (FISH)

Dual-color FISH analysis was performed on 4 µm thick tissue sections using Vysis LSI *CDKN2A* (P16) (9p21)—Spectrum Orange)/D9Z1 (9p11q11)—CEP 9 Spectrum Green probes (Abbott Molecular, Abbott Park, IL, USA). The LSI *CDKN2A* probe spans approximately 222 kb and contains several genes including MTAP, *CDKN2A*, and *CDKN2B*. The CEP 9 Spectrum Green probe hybridizes alpha satellite sequences specific to chromosome 9. Homozygous deletion was defined as having zero red signals and two green signals.

## 3. Results

### 3.1. Patient Cohort

A total of 50 patients were included in this study. There were 32 females and 18 males with a mean age at diagnosis of 53 years (Table 1). Twenty-six grade 1, 16 grade 2, and eight grade 3 meningiomas from 50 patients were evaluated in two TMAs. The mean mitotic count was 1/10 HPF for grade 1, 5/10 HPF for grade 2, and 25/10 HPF for grade 3 meningiomas evaluated on H&E-stained sections. When quantified with the sensitive Phospho-histone H3 (PHH3), the average mitotic count was 2/10 HPF for grade 1, 6/10 HPF for grade 2, and 29/10 HPF for grade 3. Average Ki67 was 3%, 9%, and 34% for grades 1, 2, and 3, respectively. All patient characteristics can be found in Supplementary Table S1. Because of variability in sections obtained from the TMAs, only 43 cases were analyzed by IHC and FISH (22, 14, and 7 cases for grades 1, 2, and 3, respectively (Table 1).

### 3.2. MTAP IHC

**Figure 1:**
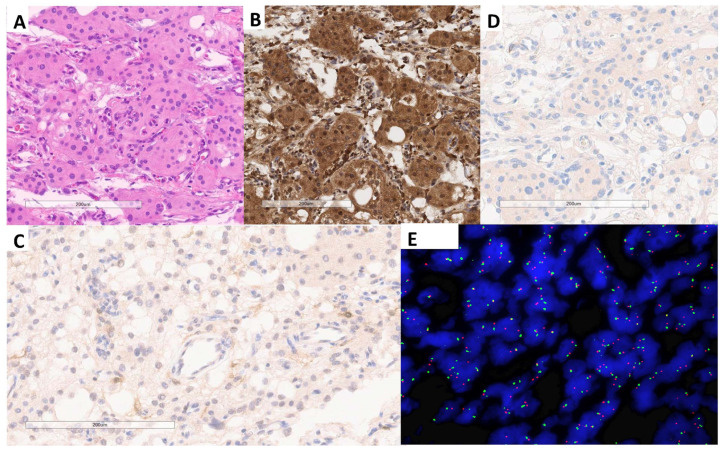
MTAP, p16, and FISH results for case #36. (**A**) H&E-stained section. (**B**) MTAP expression with 1:200 dilution at institution 1. (**C**,**D**) Faint positive p16 expression, institution 1 and institution 2, respectively. (**E**) Retained *CDKN2A* with two green and two red signals. (magnification = 600X).

**Figure 2:**
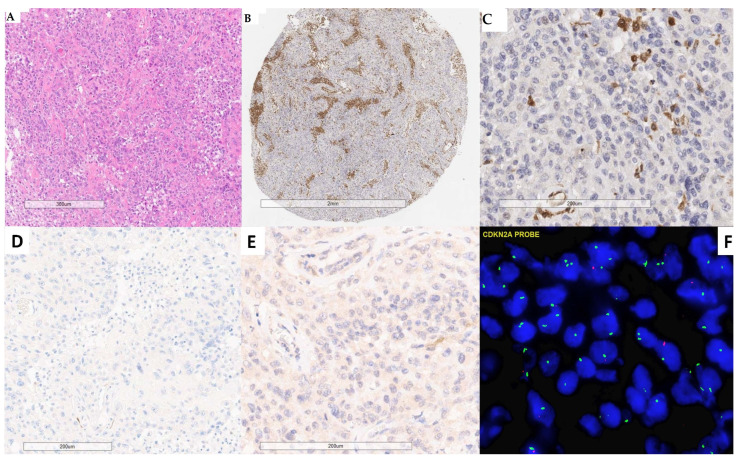
MTAP, p16, and FISH results for a grade 3 meningioma with *CDKN2A* deletion (case #47). (**A**) H&E-stained section. (**B**) Absence of MTAP expression (1:100 dilution). Tumor cells do not stain with MTAP, whereas macrophages show positive staining. (**C**) Absence of MTAP expression in tumor cells (1:100 dilution). (**D**) Absence of p16 expression in tumor cells (institution 2). (**E**) Faint staining with p16 (institution 1). (**F**) *CDKN2A* loss with the absence of red signals. (**A**–**E**) scale bars = 200 um, (**F**) magnification = 600X.

**Figure 3:**
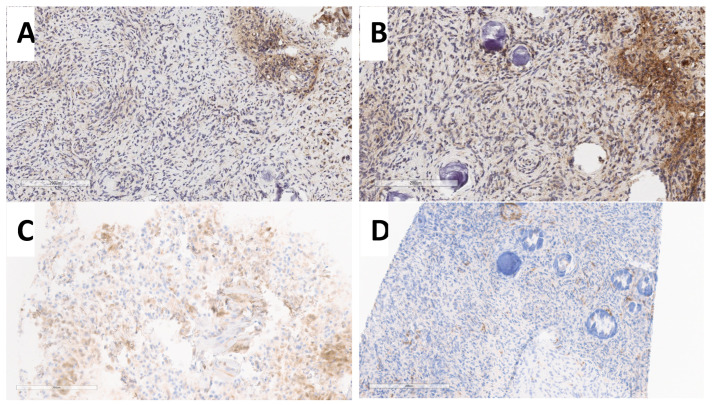

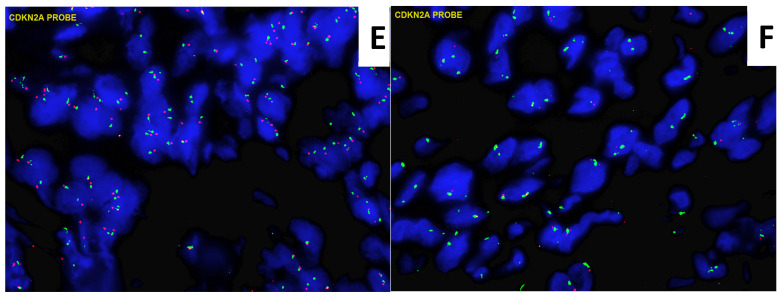
MTAP, p16, and FISH results of #30. (**A**,**B**) Grade 2 meningioma showing focal MTAP expression at 1:200 and 1:100 dilutions (institution 1), respectively. (**C**) Positive p16 expression in tumor cells. (**D**) Absence of p16 expression in tumor cells. (**E**) Areas with retained *CDKN2A* with two red and two green signals. (**F**) Area with *CDKN2A* loss with the absence of red signals in most nuclei. (**A**–**D**) scale bars = 200 um. (**E**,**F**): magnification = 600X.

**Figure 4:**
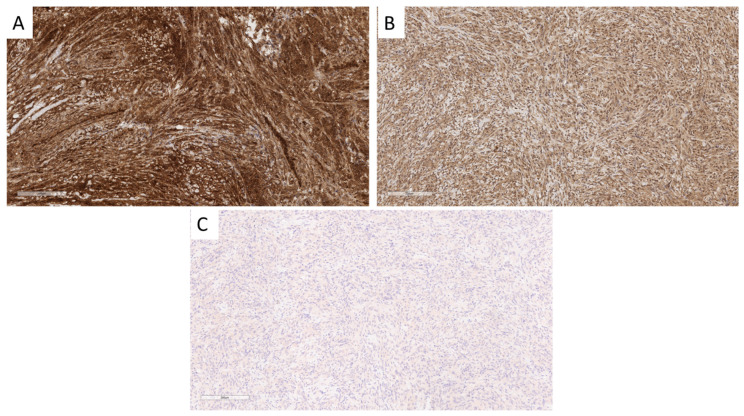
Analysis of MTAP protein expression for case #21 (grade 1 meningioma) at different dilutions. (**A**) MTAP expression at 1:100 dilution at institution 1. (**B**) MTAP expression at 1:200 dilution at institution 1. (**C**) MTAP expression at 1:1200 dilution at institution 2. Note strong expression in panels (**A**,**B**) and very faint staining in panel (**C**) Scale bars = 200 um.

**Figure 5:**
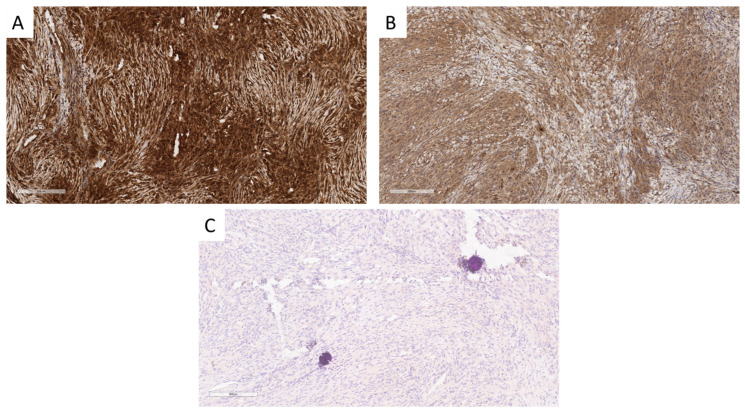
Analysis of MTAP protein expression for case #12 (grade 1 meningioma) at different dilutions. (**A**) MTAP expression with 1:100 dilution at institution 1. (**B**) MTAP expression with 1:200 dilution at institution 1. (**C**) MTAP expression with 1:1200 dilution at institution 2. Scale bars = 200 um.

MTAP IHC was performed at 1:400, 1:200, and 1:100 dilutions at institution 1 and 1:1200 at institution 2. (Table 2). The results from institution 1 showing MTAP expression of a grade 2 meningioma (#36) can be seen in Figure 1. Thirty-two (32/43) tumors showed expression of MTAP (1:400 dilution). Forty-two (42/43) tumors showed expression of MTAP at the 1:200 dilution, with one meningioma showing loss of MTAP expression (#47, Figure 2) and two cases showing focal expression (#19 and #30, Figure 3). When the MTAP antibody concentration was increased to 1:100, case #19 showed MTAP expression, whereas case #30 continued to show focal expression. Case #47 showed absence of MTAP expression in 1:100 dilution as well. (Figure 2). Analysis of MTAP expression at institution 2 at the 1:1200 dilution indicated that sixteen (16/43) tumors showed expression of MTAP, twenty-three (23/43) showed very faint positive expression, and four (4/43) showed absence of MTAP expression. (Table 2). The results of case #21 and case #12 at institution 1 (at dilutions of 1:100 and 1:200) and institution 2 (at the dilution of 1:1200) are shown in Figure 4 and Figure 5, respectively.

### 3.3. p16 IHC

For institution 1, 41/43 cases showed p16 expression as follows: 22 positives, 18 faint positives, and one focal positive (#30), and two were negative for p16 expression (#5 and #27). For institution 2, 32/43 meningiomas showed p16 expression as follows: 21 positives, 10 faint positives, and one focal positive (#30), and 11 were negative for p16 expression.

### 3.4. CDKN2A FISH

**Figure 6:**
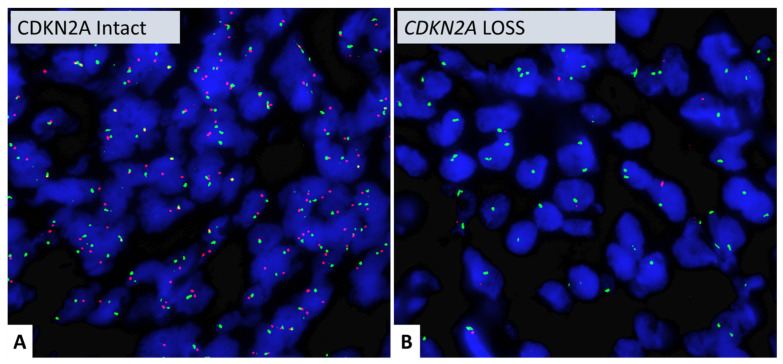
Vysis LSI *CDKN2A* (P16) (9p21)—Spectrum Orange)/D9Z1 (9p11q11)—CEP 9 Spectrum Green probes (Abbott Molecular, Abbott Park, IL, USA). (**A**) *CDKN2A* intact tumor with two red and two green signals. (**B**) Tumor with loss of red signals but presence of two green signals, indicating homozygous loss of *CDKN2A*. Magnification = 600X.

CDKN2A FISH analysis was performed on 43 meningiomas in a CLIA-certified CAP-accredited laboratory (Table 2). Forty-one (41/43) cases showed two green and two red signals, indicating two copies of *CDKN2A* and two copies of chromosome 9. One (1/43) case showed loss of both red signals with preservation of the two green signals, consistent with homozygous *CDKN2A* deletion (case #47). The other case (#30) showed focal loss of both red signals, consistent with focal *CDKN2A* deletion (Figure 6).

Case #47 showed loss of red signals by FISH and negative staining with MTAP 1:1200 (institution 2), 1:400, 1:200, and 1:100 dilutions (institution 1) (Figure 2). P16 was negative in institution 2, but faint positive staining was observed in institution 1 (Figure 2).

In contrast, case #30 showed focal loss of red signals with the FISH assay and had negative staining with MTAP 1:1200 and 1:400 dilutions, but focal positive staining at the 1:200 and 1:100 dilutions. P16 showed focal expression by IHC staining performed at both institutions, concordant with the MTAP results (Table 2) (Figure 3).

## 4. Discussion

In this study, we investigated the utility of MTAP and p16 IHC as surrogate markers of *CDKN2A* deletion in meningiomas. This is clinically relevant given that homozygous deletion of CDKN2A/B is part of the criteria that determine the WHO grade of meningiomas under the most recent WHO Classification of CNS tumors [2]. Homozygous *CDKN2A* deletions occur in approximately 4.9% of meningiomas [12]. We examined the expression of MTAP at three different dilutions, i.e., 1:400, 1:200, and 1:100, at institution 1 and a 1:1200 dilution at institution 2, and we examined p16 expression using the same antibody clone but with different staining conditions at 2 different CLIA-accredited and CAP-certified laboratories. *CDKN2A* FISH was used as the gold standard for evaluating *CDKN2A* status. Forty-three cases were successfully analyzed by FISH and 41/43 (95%) showed intact *CDKN2A* status.

FISH studies revealed one grade 3 meningioma with homozygous *CDKN2A* loss (2%) and one grade 2 meningioma with focal *CDKN2A* loss (2%). In our study, we observed a focal loss of *CDKN2A* in 7% of grade 2 cases (1 out of 14) and a homozygous deletion of *CDKN2A* in 14% of grade 3 cases (one out of seven).

These percentages are smaller than a recently published study by Sasaki et al., which identified 17% of grade 2 meningiomas with *CDKN2A* loss by FISH [12% (3/26) and 67% (2/3) of grade 3] [11]. All 41 cases with intact *CDKN2A* by FISH showed MTAP expression (1:200 and 1:100 dilutions) at institution 1. Similarly, thirty-nine cases (39/41) at institution 2 showed MTAP expression. The two cases with complete or partial *CDKN2A* deletion by FISH showed loss of MTAP expression by IHC performed at both institutions. These results indicate that MTAP expression can serves as a reliable surrogate marker of *CDKN2A* status in meningiomas at dilutions of 1:200 and 1:100 (antibody; Abnova Anti-MTAP Monoclonal (2G4)), but its sensitivity diminishes with a different antibody at a dilution of 1:1200 (antibody; Protein Tech Anti-MTAP Polyclonal).

In contrast to the excellent correlation between MTAP expression at dilutions of 1:200 and 1:100 and *CDKN2A* status, there was a poor correlation between p16 expression and *CDKN2A* status. Only 20/41 (48%) of meningiomas with intact *CDKN2A* status showed expression of p16. The two meningiomas with partial and complete loss of *CDKN2A*, also showed partial and complete loss of p16 expression, respectively.

In our study, MTAP expression showed 100% (41/41) specificity at the 1:100 dilution and 95% (39/41) specificity at the 1:1200 dilution for identifying meningiomas with intact *CDKN2A* status and 100% (2/2) sensitivity for detecting meningiomas with *CDKN2A* homozygous loss. For anti-MTAP at a dilution of 1:200 using the Abnova antibody, we observed focal loss in approximately 5% of cases (2/43) and a negative result in 2% of cases (1/43). When compared with *CDKN2A* FISH results, we observed focal loss in 2% of cases (1/43) and homozygous deletion in 2% of cases (1/43). This indicates that evaluation of MTAP expression by IHC at the conditions indicated above demonstrated 100% sensitivity (2/2) and 98% specificity (40/41) for evaluating *CDKN2A* status in meningiomas This result aligns with the findings of Sasaki et al., who reported that the loss of MTAP expression showed 100% sensitivity (5/5) and 100% specificity (24/24) for detecting *CDKN2A* homozygous deletion using the same anti-MTAP at the same dilution of 1:200 (10). In addition, we evaluated the correlation between p16 expression and *CDKN2A* status and MTAP expression. For both institutions, the sensitivity of the absence of p16 immunostaining for identifying meningiomas with *CDKN2A* homozygous loss was 50% (1/2). p16 staining differences between the two institutions resulted in different specificities. For institution 1, the specificity to detect intact *CKDN2A* was 95% (39/41), with 17 of these tumors showing faint positive staining; for institution 2, the specificity to detect intact *CKDN2A* was 76% (31/41), with 10 cases showing faint expression. Tang et al. recently reported a p16 IHC specificity of 90% in grades 2 and 3 meningiomas for detecting *CDKN2A* homozygous deletion [13]. They also found a poor correlation between p16 IHC and *CDKN2A* status in grade 1 meningiomas. In our study, even though institution 1 showed high sensitivity and specificity for p16, given the inconsistency between the institutions and the high amount of faint positive staining, p16 alone might not be as good as MTAP for determining CDKN2A/B status in meningiomas.

In our study, at institution 1 there were two meningiomas with focal MTAP expression at the 1:200 dilution (one grade 1 and two grade 2 meningiomas). The grade 1 meningioma (#19), which showed intact *CDKN2A* status by FISH, showed expression of MTAP when the antibody concentration was increased to a 1:100 dilution. The grade 2 meningioma (#30) maintained focal MTAP expression even at the higher antibody concentration (1:100). Case #30 showed focal *CDKN2A* loss, consistent with the focal MTAP expression. At institution 2, among the 43 cases subjected to FISH analysis, MTAP expression at the 1:1200 dilution exhibited expression in sixteen (16/43) tumors, faint positive expression in twenty-three cases (23/43), and an absence of MTAP expression in four cases (4/43). Notably, among these four cases lacking MTAP expression, case #47 (grade 3) and case #30 exhibited complete *CDKN2A* loss and focal loss of *CDKN2A*, respectively, as confirmed by FISH, aligning with the MTAP IHC results. Conversely, cases #44 and #39 demonstrated positive results for *CDKN2A* on FISH and loss of MTAP expression. These findings underscore the reliability of MTAP expression as a surrogate marker for *CDKN2A* status, particularly at a 1:100 dilution with the Abnova Anti-MTAP antibody, but it is not as sensitive at a dilution of 1:1200 with the Proteintech Anti-MTAP antibody given the significant number of faint positive results.

Several studies have investigated MTAP IHC as a marker of *CDKN2A* status in various tumor types such as diffuse glioma [14], pleomorphic xantroastrocytoma [15], and mesothelioma [9,16,17]. Satomi et al. investigated MTAP immunostaining as a surrogate marker for *CDKN2A* loss in 178 diffuse gliomas (77 astrocytoma IDH-mutant, 13 oligodendrogliomas, and 88 glioblastomas, IDH-wildtype) [2,14]. They reported that MTAP can identify *CDKN2A* status with 88% sensitivity and 98% specificity in astrocytoma Isocitrate dehydrogenase (IDH) mutant tumors, and 89% sensitivity and 100% specificity in glioblastoma, IDH-wildtype. The sensitivity and specificity observed were lower in oligodendrogliomas (67% and 57%, respectively).

Chapel et al. identified MTAP immunostaining as a surrogate marker for homozygous *CDKN2A* deletion, with 80% sensitivity and 100% specificity [18]. They also showed that MTAP has high interobserver consistency and reproducibility among the different laboratories. Lou et al. evaluated MTAP staining in a total of 23 pleomorphic xanthoastrocytomas and compared MTAP expression against *CDKN2A* FISH [15]. Their results showed a sensitivity of 86.7% and specificity of 100% for MTAP as a surrogate marker for identifying *CDKN2A* status. They did observe, however, MTAP immunopositivity in two tumors that showed *CDKN2A* homozygous deletion by FISH (using a ~345 kb FISH probe). Because this probe covers a large region from MTAP to p15INK4B, *CDKN2A* FISH results can also represent the deletions in other genes such as p16, MTAP, and p15INK4B that may interfere with the binding of the larger probe [19]. Thus, even though two pleomorphic astrocytomas had a result of homozygous deletion of *CDKN2A* by FISH, MTAP was expressed on IHC. Several antibodies are routinely employed in clinical practice as surrogate markers for detecting mutations in specific genes associated with various brain tumors. For example, antibodies against ATRX, K27me3, and INI1 serve as reliable indicators of mutations in the corresponding genes. ATRX loss is frequently used to diagnose gliomas, loss of H3K27me3 is employed to identify specific subtypes of diffuse midline gliomas, and INI1 loss is characteristic of atypical teratoid/rhabdoid tumors. These markers provide a cost-effective and accessible alternative for the molecular classification and diagnosis of various brain tumors [20,21].

Among 41 meningiomas with intact *CDKN2A*, 22 of them showed p16 expression, 17 showed faint expression at institution 1, whereas at institution 2, 21 cases showed expression and 10 cases showed faint expression. The p16 results did not correlate with either *CDKN2A* FISH results or MTAP immunostaining. However, in a recent study that was conducted on 56 gliomas, 38 cases showed no expression with p16 and *CDKN2A* homozygous deletion with FISH [22]. The remaining 28 gliomas had intense and diffuse p16 expression and did not show *CDKN2A* homozygous deletion. Our results with p16 expression in meningiomas show limited correlation with *CDKN2A* status, suggesting that MTAP immunostaining is superior to p16 for the evaluation of CDKN2A/B deletion in meningiomas.

## 5. Conclusions

MTAP immunostaining can be a good surrogate marker for identifying *CDKN2A* homozygous deletion in meningiomas. Focal MTAP expression meningiomas can be assessed with *CDKN2A* FISH to determine true *CDKN2A* status. MTAP expression is more sensitive at a dilution of 1:100 with the Abnova Anti-MTAP Monoclonal antibody. One limitation of using MTAP IHC as a surrogate marker for *CDKN2A* homozygous deletion lies where the deletion is sufficiently small, affecting solely the *CDKN2A* gene. In such cases, it may not encompass the MTAP gene, thereby leaving MTAP expression unaffected even in cases of *CDKN2A* loss. P16 appears to be a less reliable marker of *CDKN2A* status in meningiomas.

## Figures and Tables

**Table 1:** Clinicopathologic characteristics of MTAP-positive and -negative tumors (MTAP dilution 1:100).

	Total (*n* = 50)	MTAP Pos (*n* = 41)	MTAP Focal Pos (*n* = 1)	MTAP Neg (*n* = 1)
Age at diagnosis (y)	53 ± 14	53 ± 13	80	51
Female	32 (64%)	26 (63%)	1(100%)	1(100%)
WHO grade				
Grade 1	26	22 (54%)	0	0
Grade 2	16	13 (32%)	1(100%)	0
Grade 3	8	6 (15%)	0	1 (100%)
Mean mitotic count (H&E)	5.5	4.5	5	20
Mean mitotic count (PHH3)	7.5	7.2	6	36
Ki67 average (%)	9.8	9.3	6.5	31
Ki67 maximum (%, mean)	11.3	11.2	8.5	-
*CDKN2A* HD	1	41 (100%)	1 (100%)	1(100%)

**Table 2:** MTAP and p16 immunohistochemistry results from both institutions at different dilutions. Abbreviations: pos: positive, neg: negative, Inst: institution, N/A: not applicable.

Patient ID	Histologic Grade	MTAP (1:400) (Inst 1)	MTAP (1:200) (Inst 1)	MTAP (1:100) (Inst 1)	MTAP (1:1200) (Inst 2)	P16 (Inst 1)	P16 (Inst 2)	*CDKN2A* FISH
1	1	pos	pos	pos	faint pos	pos	pos	retained
2	1	neg	pos	pos	faint pos	pos	pos	retained
3	1	neg	pos	pos	faint pos	faint pos	neg	retained
4	1	neg	pos	pos	faint pos	faint pos	neg	retained
5	1	neg	pos	pos	faint pos	neg	neg	retained
6	1	pos	pos	pos	faint pos	pos	faint pos	retained
7	1	neg	pos	pos	faint pos	pos	pos	N/A
8	1	pos	pos	pos	faint pos	pos	pos	retained
9	1	pos	pos	pos	faint pos	pos	neg	retained
10	1	pos	pos	pos	faint pos	pos	pos	retained
11	1	pos	pos	pos	faint pos	pos	pos	retained
12	1	pos	pos	pos	faint pos	pos	pos	retained
13	1	N/A	N/A	pos	faint pos-	N/A	N/A	N/A
14	1	neg	pos	pos	faint pos	pos	pos	retained
15	1	neg	pos	pos	N/A	neg	neg	N/A
16	1	pos	pos	pos	faint pos	pos	faint pos	retained
17	1	pos	pos	pos	faint pos	faint pos	neg	retained
18	1	neg	pos	pos	faint pos	faint pos	neg	retained
19	1	neg	focal pos	pos	faint pos	faint pos	faint pos	retained
20	1	pos	pos	pos	faint pos	pos	pos	retained
21	1	pos	pos	pos	faint pos	faint pos	pos	retained
22	1	pos	pos	pos	faint pos	faint pos	faint pos	retained
23	1	neg	pos	pos	faint pos-	pos	pos	N/A
24	1	pos	pos	pos	faint pos	pos	pos	retained
25	1	pos	pos	pos	faint pos	pos	pos	retained
26	1	pos	pos	pos	pos	faint pos	faint pos	retained
27	2	pos	pos	pos	pos	neg	neg	retained
28	2	pos	pos	pos	pos	faint pos	pos	retained
29	2	N/A	N/A	pos	neg-	pos	pos	N/A
30	2	neg	focal pos	focal pos	neg	focal pos	focal pos	focal loss
31	2	pos	pos	pos	pos	faint pos	neg	retained
32	2	pos	pos	pos	faint pos	pos	pos	retained
33	2	pos	pos	pos	pos	faint pos	faint pos	retained
34	2	pos	pos	pos	pos	faint pos	neg	retained
35	2	N/A	N/A	pos	pos-	N/A	N/A	N/A
36	2	pos	pos	pos	pos	pos	faint pos	retained
37	2	pos	pos	pos	faint pos	pos	pos	retained
38	2	pos	pos	pos	pos	pos	pos	retained
39	2	neg	pos	pos	neg	faint pos	faint pos	retained
40	2	pos	pos	pos	pos	pos	pos	retained
41	2	pos	pos	pos	pos	faint pos	pos	retained
42	2	pos	pos	pos	pos	faint pos	faint pos	retained
43	3	pos	pos	pos	pos	pos	pos	retained
44	3	pos	pos	pos	neg	faint pos	faint pos	retained
45	3	pos	pos	pos	pos	pos	pos	retained
46	3	pos	pos	pos	pos	faint pos	neg	retained
47	3	neg	neg	neg	neg	faint pos	neg	loss
48	3	neg	pos	pos	pos	pos	pos	retained
49	3	pos	pos	pos	pos	pos	pos	retained
50	3	N/A	N/A	N/A	pos	N/A	N/A	N/A

## Data Availability

The original contributions presented in this study are included in this article/Supplementary Materials. Further inquiries can be directed to the corresponding authors.

## Supplementary Materials

The following supporting information can be downloaded at https://www.mdpi.com/article/10.3390/cancers16193299/s1: Table S1. Clinicopathological characteristics of all meningioma patients.
