# Supplementary material for: MTAP and p16 IHC as Markers for CDKN2A/B Loss in Meningiomas
Source: Cancers (Basel). 2024 Sep 27;16(19):3299. doi: 10.3390/cancers16193299 (PMC11476088; doi:10.3390/cancers16193299)
Supplement: Supplementary file 1 [file cancers-16-03299-s001.zip › cancers-3197496-supplementary.pdf]

Table S1. Clinicopathological characteristics of all meningioma patients.

Patient ID	Age at Diagnosis, Years	Race	Sex	Histologic grade	Mitosis (10 HPF) (PHH3)	Progression	PFS (months)	Death	OS (months)	Location
1	44	White or Caucasian	F	1	1	0	1.58	0	1.58	Left frontal convexity
2	41	Black or African American	F	1	3	0	8.12	0	8.12	Falx cerebri
3	52	White or Caucasian	F	1	N/A	0	57.83	0	57.83	Right posterior fossa tumor
4	38	Black or African American	F	1	2	0	80.22	0	80.22	Right skull base
5	39	White or Caucasian	F	1	0	0	42.41	0	42.41	Olfactory groove/Anterior skull base
6	26	Other	F	1	1	0	69.30	0	69.30	Right lateral ventricle
7	73	White or Caucasian	F	1	5	0	36.62	0	36.62	Anterior skull base
8	52	Unknown	F	1	2	0	5.26	0	5.26	Tuberculum sella/Skull base
9	77	White or Caucasian	F	1	3	0	41.52	0	41.52	Right sphenoid wing/Middle cranial fossa/Right temporal skull base
10	53	White or Caucasian	M	1	1	0	58.75	0	58.75	Right posterior fossa
11	61	White or Caucasian	F	1	0	0	0.66	0	0.66	Tuberculum sella/Skull base
12	55	White or Caucasian	M	1	1	0	2.60	0	2.60	Left frontal
13	43	Other	F	1	3	0	70.29	0	70.29	Tuberculum sella/Suprasellar region
14	50	Asian	M	1	1	0	60.56	0	60.56	Left frontal
15	39	Black or African American	M	1	1	0	56.68	0	56.68	Sellar/Suprasellar/Skull base
16	44	White or Caucasian	M	1	N/A	1	63.22	0	79.92	Left posterior orbital mass
17	57	Other	F	1	2	0	63.78	0	63.78	Right posterior fossa
18	57	White or Caucasian	F	1	3	0	70.09	0	70.09	Parietal
19	60	Black or African American	F	1	1	0	60.16	0	60.16	Left foramen magnum/Left skull base
20	54	White or Caucasian	F	1	5	0	13.91	0	13.91	Skull base
21	63	White or Caucasian	F	1	3	0	60.56	0	60.56	Right skull base/Olfactory groove
22	39	White or Caucasian	M	1	3	0	60.99	0	60.99	Right skull base
23	36	White or Caucasian	M	1	4	0	18.31	0	18.31	Left frontal
24	33	Other	F	1	N/A	0	65.82	0	65.82	Right parasagittal
25	48	White or Caucasian	M	1	3	0	64.54	1	64.54	Right lateral ventricle
26	58	White or Caucasian	F	1	N/A	0	12.36	0	12.36	Right skull base tumor
27	40	White or Caucasian	F	2	2	0	11.08	0	11.08	Left falx
28	54	Asian	F	2	11	0	70.19	0	70.19	Right frontal region
29	53	Black or African American	M	2	N/A	1	65.95	0	130.65	Cranial meninges
30	80	Other	F	2	6	0	60.82	0	60.82	Spinal meninges (level T1-T3)
31	81	White or Caucasian	M	2	8	0	67.46	0	67.46	Left skull base
32	55	White or Caucasian	M	2	N/A	0	36.76	1	36.76	Left frontal lobe
33	37	White or Caucasian	F	2	6	0	73.71	0	73.71	Left sphenoid wing
34	34	White or Caucasian	M	2	6	0	48.66	0	48.66	Left frontoparietal region
35	48	Asian	F	2	N/A	0	51.52	0	51.52	Left frontal
36	54	White or Caucasian	M	2	8	0	60.07	0	60.07	Left frontal
37	76	Other	M	2	N/A	0	66.58	0	66.58	Left frontal
38	54	White or Caucasian	M	2	6	1	76.34	0	128.32	Left frontal, left parietal, left falx
39	30	White or Caucasian	M	2	5	0	25.58	0	25.58	Right parietal
40	59	White or Caucasian	F	2	10	1	37.78	0	75.91	Left ventricle

41	70	Black or African American	F	2	5	0	76.80	0	76.80	Right temporal
42	56	White or Caucasian	F	2	5	0	71.41	0	71.41	Left temporal convexity
43	36	White or Caucasian	F	3	18	1	5.29	1	10.98	Occipital
44	75	Black or African American	M	3	N/A	0	27.42	1	27.42	Right frontal falcine, right frontal temporal
45	77	White or Caucasian	M	3	28	0	55.20	0	55.20	Right parietal
46	54	White or Caucasian	F	3	38	0	13.28	0	13.28	Right suboccipital soft tissue
47	51	White or Caucasian	F	3	36	1	40.04	0	105.17	Right frontal lobe
48	61	White or Caucasian	F	3	25	1	62.17	0	93.93	Parietal parasagittal
49	56	White or Caucasian	F	3	28	0	30.97	0	30.97	Left posterior fossa
50	76	White or Caucasian	F	3	N/A	1	4.01	0	8.78	Left occipital
